# Immune and inflammatory pathways in NASH

**DOI:** 10.1007/s12072-013-9468-6

**Published:** 2013-08-30

**Authors:** Michal Ganz, Gyongyi Szabo

**Affiliations:** Department of Medicine, University of Massachusetts Medical School, LRB208, 364 Plantation Street, Worcester, MA 01605 USA

**Keywords:** NAFLD, NASH, Inflammation, PAMPs, DAMPs, Immunity

## Abstract

Immune and inflammatory pathways have a central role in the pathogenesis of non-alcoholic fatty liver disease (NAFLD). Both the innate and adaptive immune systems contribute to the development of NAFLD. Pathogen-associated molecular patterns and danger-associated molecular patterns are known to activate a variety of pattern-recognition receptors that result in inflammation. The key features of the immune system and inflammatory pathways in the development of NAFLD are discussed in this review.

## Introduction

Non-alcoholic fatty liver disease (NAFLD) is the most common liver disease worldwide, affecting around one third of the Western world with an incidence that continues to grow even in other parts of the world [1, 2]. NAFLD includes a variety of histopathological findings ranging from simple steatosis with no inflammation to steatosis with varying degrees of inflammation (steatohepatitis, NASH) to cirrhosis [1]. It is thought that around 10–20 % of patients with NAFLD have NASH [3]. Patients with NASH have the potential to develop fibrosis and cirrhosis leading to portal hypertension, liver decompensation, and hepatocellular carcinoma [4].

The liver is composed of a diverse array of cell types, most of which have the potential to be involved in inflammation. Hepatocytes make up around 60–80 % of all liver cells, and they are responsible for the metabolic, biosynthetic, biliary secretion, and detoxification roles of the liver. The liver is also enriched in various immune cells. The resident innate immune cells in the liver are comprised of Kupffer cells (KCs), the resident macrophages, and natural killer (NK) cells. Upon liver injury, other innate immune cells, including neutrophils, leukocytes, monocytes, and inflammatory macrophages, are rapidly recruited to the liver. The adaptive immune cells consist primarily of natural killer T (NKT) cells, B cells, and T cells. Other cell types that may contribute to inflammation and fibrosis are the sinusoidal endothelial cells (SECs) and hepatic stellate cells (HSCs).

The cells in the liver contribute to the inflammation that results in the development and progression of steatohepatitis, which is secondary to the immune response of the liver to danger signals, likely from a combination of exogenous and endogenous danger signals, which include injured self-molecules, i.e., DNA and RNA. There are multiple “hits” needed for NASH to develop and progress. In addition to metabolic factors, innate immune alterations, including inflammation caused by free fatty acids (FFA), bacterial lipopolysaccharide (LPS), chemokines, cytokines, and adipokines, all contribute [3].

## Contribution of immune cells

Innate immune cells, consisting of KCs, neutrophils, dendritic cells (DCs), and NK cells, all play a role in the pathogenesis of NASH. In the setting of acute or chronic liver disease, KCs are activated by both pathogen-associated molecular patterns (PAMPs) and damage-associated molecular patterns (DAMPs). This activation results in the release of proinflammatory cytokines, such as tumor-necrosis factor α (TNFα), interleukin-6 (IL-6), and interleukin-1β (IL-1β), which lead to T cell activation and induction of apoptosis and HSCs [5]. There are two types of macrophages, M1 or “classically activated” macrophages, which play an important role in humoral immunity and response to pathogens, and M2 or “alternatively activated” macrophages, which have anti-inflammatory properties. There is an increase in M1 relative to M2 macrophages in adipose tissue and in the liver in response to high-fat diet (HFD) feeding and obesity [6–8]. The imbalance between M1 and M2 macrophages may have an important role in the pathogenesis of NASH. Depletion of KCs attenuates methionine-choline-deficient (MCD) and HFD-induced steatohepatitis [9, 10].

Activated neutrophils release proinflammatory cytokines and myeloperoxidase, resulting in oxidative damage to hepatocytes [11, 12]. Furthermore, an increase in the ratio of neutrophils to lymphocytes can increase the likelihood of NASH progression [13]. DCs also appear to play an important role in NASH, as recent reports have shown that DC depletion results in worsening NASH severity, suggesting a regulator role of DCs in NASH [14].

NK cells are an important component of the immune system and play a role in linking the innate and adaptive immune responses [15]. NK cells are abundant in the liver, and in disease they have a role in the development of liver injury and fibrosis. It is thought that NK cells could play a role in the development of NAFLD/NASH. NK cell-activating ligands are increased in human NASH patients and in mice following an MCD diet [16]. Obese patients have both a decrease in circulating levels and impaired cytotoxic function of NK cells as compared to lean controls [17]. Similar impairment in cytotoxic activity was determined to be present in rats with HFD-induced obesity [18]. NK cells may have an anti-fibrotic effect in the liver [19], and the reduction in their levels and activity may contribute to the increased susceptibility of obese patients to develop cirrhosis.

Natural killer T (NKT) cells are a subset of lymphocytes that express NK cell markers, such as CD161 and CD94, as well as a T cell receptor. NKT cells exhibit features of both innate and adaptive immune cells and act as a bridging system between innate and adaptive immunity [20]. NKT cells are enriched within the liver and regulate the immune response by secreting both T_H_1 and T_H_2 cytokines after stimulation [21]. Biopsies in patients with NAFLD showed that NKT cell population was decreased when there was moderate to severe steatosis [22]. Both obese leptin-deficient ob/ob mice and HFD-induced obesity showed reduced liver NKT cells [23, 24]. Upregulation of hepatic NKT cells results in improvement of HFD-induced hepatic steatosis [25]. Depletion of NKT cells leads to chronic inflammatory conditions that contribute to hepatic steatosis [23]. On the other hand, there is an increase of NKT cells in MCD diet-induced steatohepatitis and in cirrhotic livers [26]. NKT cells appear to drive production of osteopontin and of hedgehog ligands leading to fibrogenesis, which may explain the increase in NKT cells in the MCD diet model and in cirrhotic livers [27].

Regulatory T cells (T_regs_) are a subset of T cells that are either naturally occurring or inducible. An important role of the CD25+ subset of T_regs_ is their suppression of CD4 and CD8 T cells and thus in a decrease in inflammation. Mouse models have displayed that depleting CD25+ T_regs_ results in precipitation of autoimmune disease. In the liver, it has been reported that there are increased T_regs_ in tumor tissue in HCC patients and in patients with either chronic HBV or HCV infection [28–30], suggesting that T_regs_ have a role in either suppressing or maintaining tumor cells or viral infection. In NASH livers, there are increased numbers of T_regs_ [31, 32]. Mice fed a HFD showed a gradual decrease of T_regs_ over time, which may be a key event in the progression from steatosis to steatohepatitis, as T_regs_ may help control hepatic inflammation [33].

T helper type 17 cells (T_H_17 cells) are another subset of T cells that produce interleukin-17 (IL-17) and mediate various immune responses. T_H_17 cells play a role in mediating pathogen clearance and in inflammatory responses in tissues. Studies have shown the importance of T_H_17 cells in autoimmune liver diseases, chronic viral hepatitis, alcoholic liver disease, and hepatocellular carcinoma [34–36]. Tang et al. [37] show that there are increased numbers of T_H_17 cells in the liver after an 8-week HFD feeding and that these cells are associated with the progression of hepatic steatosis and inflammation through the production of IL-17. The same group suggests that the balance between T_H_17 cells and T_regs_ could play a role in the progression of NASH; however, further studies need to be done to answer this question.

## Pathogen-associated molecular patterns (PAMPs)

**Fig. 1:**
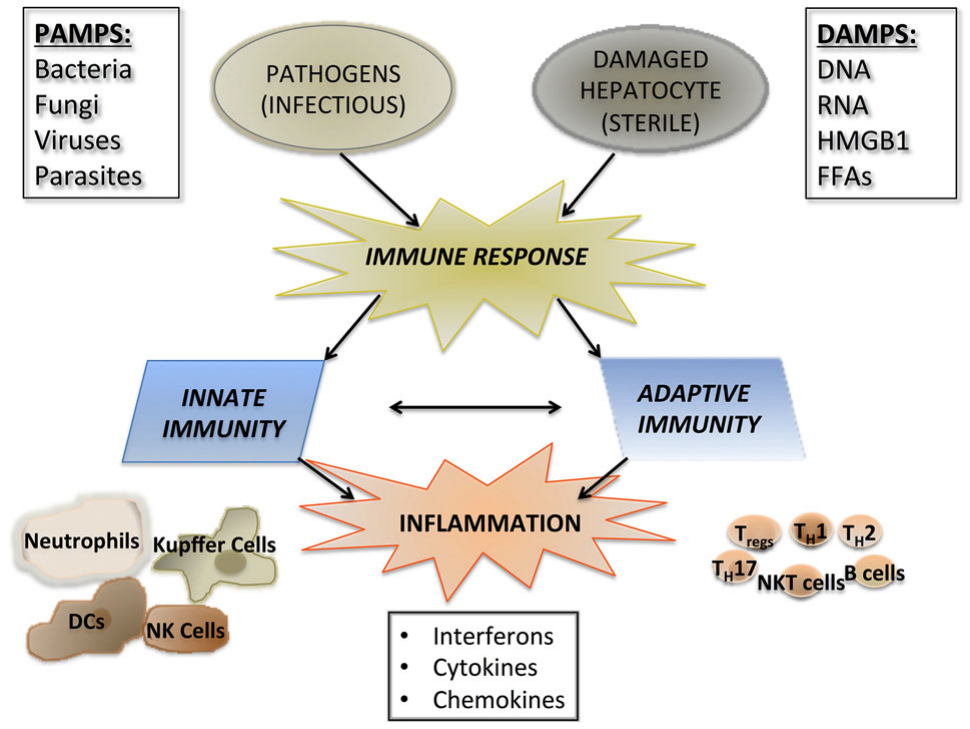
Immune response in NASH. The immune response in NASH is initiated by both pathogen-associated molecular danger signals and danger-associated molecular signals. This response involves both the innate and adaptive branches of the immune system and results in inflammation

**Fig. 2:**
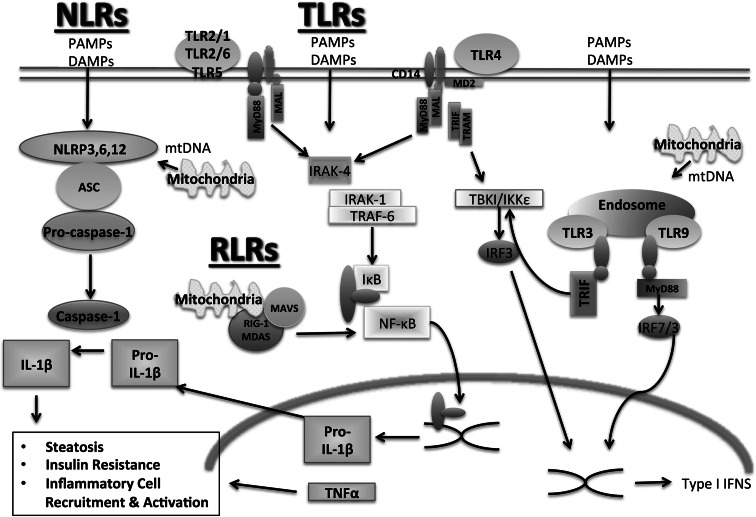
Pattern recognition receptors in NASH. A variety of pattern-associated molecular patterns and danger-associated molecular patterns are involved in the pathogenesis of NASH. PAMP and DAMP signaling can occur through various PRRs, including Toll-like receptors, nucleotide-binding and oligomerization domain (NOD)-like receptors, and RIG-I like receptors. PRR signaling can result in steatosis, insulin resistance, and inflammatory cell activation and recruitment

PAMPs are exogenous danger signals derived from microbes that result in inflammation. PAMPs are known to contribute to inflammation in NASH by signaling through PRRs, leading to the activation of both the innate and adaptive arms of the immune system (Fig. 1). PAMPs are recognized by a diverse array of pattern recognition receptors (PRRs), including Toll-like receptors (TLRs), nucleotide-binding and oligomerization domain (NOD)-like receptors (NLRs), RIG-I like receptors (RLRs), and other non-specific sensors, e.g., C-type lectin receptors (CLRs) [38, 39] (Fig. 2). PAMPs are expressed by bacteria, viruses, parasites, and fungi, and they include lipids, lipoproteins, nucleic acids, and proteins [40].

### Toll-like receptors (TLRs)

There are 10 and 13 known TLRs in humans and mice, respectively. In the liver, TLRs are expressed on KCs, hepatocytes, SECs, and HSCs. TLRs recognize a wide variety of PAMPs and are found in different cellular locations: TLRs 2, 4, 5, 6, and 11 are found on extracellular surfaces, whereas TLRs 3, 7, 8, and 9 are expressed exclusively in intracellular vesicles, such as endosomes, lysosomes, and the endoplasmic reticulum [39]. Once a molecular pattern has been recognized by the TLR, downstream signaling is initiated, resulting in inflammatory and antiviral responses. Each TLR is associated with a different set of adaptor proteins. For example, the adaptor myeloid differentiation factor 88 (MyD88) is shared by all TLRs, except TLR3, and leads to recruitment of the IL-1 receptor (IL-1R)-associated kinase family. TLR signaling results in the activation of transcription factors nuclear factor kappa B (NF-κB), activator protein-1 (AP-1), or interferon regulatory factors, leading to the transcription of inflammatory cytokines, chemokines, or type I interferons, respectively.

#### TLR2/TLR1/TLR6

TLR2 recognizes a variety of PAMPs from bacteria, fungi, parasites, and viruses. These include bacterial lipoprotein, peptidoglycan, and lipoteichoic acid [41]. Dimerization of TLR2 with TLR1 or TLR6 provides additional specificity in ligand recognition. TLR2 deficiency has been shown to accelerate MCD diet-induced steatohepatitis and fibrosis [42, 43]. However, another group found that TLR2-deficient mice fed a choline-deficient amino acid (CDAA) diet had similar steatosis to wild-type mice, but decreased inflammatory cell infiltration and hepatocyte ballooning, suggesting that while steatosis is independent of TLR2 signaling, TLR2 appears to play a role in inflammation [44]. The same group showed that a TLR2 ligand activates both KCs and HSCs in vitro, but in vivo it was determined that KCs were responsible for TLR2-mediated liver inflammation and fibrosis. TLR2 knockout mice fed a HFD showed improvement in glucose tolerance and a decrease in steatosis [45]. The differences seen between the various studies could be secondary to different diets that induce steatohepatitis, as the CDAA and HFD diet result in weight gain and insulin resistance, which is not seen in the MCD diet. Another possibility is differences in intestinal microflora between the mice that could be a source of TLR2 ligands.

#### TLR3

TLR3 is present exclusively in intracellular vesicles, such as endosomes and lysosomes, and it recognizes double-stranded RNA (dsRNA) and poly(I:C), a synthetic analog of dsRNA, along with genomic RNA of a variety of other viruses [40]. Activation of TLR3 results in type I interferon and inflammatory cytokine production and thus produces an antiviral immune response. TLR3 activation has been implicated in a number of autoimmune diseases, including type I diabetes mellitus and Hashimoto’s thyroiditis [46, 47]. In NAFLD, Wu et al. [48] showed that TLR3 knockout mice fed a HFD displayed an improvement in glucose tolerance and an improvement in lipid and cholesterol metabolism, with no protection from HFD-induced obesity. It is yet to be established what the ligand leading to TLR3 activation is in NAFLD.

#### TLR4

TLR4 is the most widely studied TLR, and it recognizes a diverse array of PAMPs that include LPS, *Pseudomonas* exoenzyme, taxol, and other components of microbes [41]. TLR4 signaling occurs in a slightly different manner than in the other TLRs, as it requires an accessory molecule, glycoprotein MD2, to effectively bind LPS. Signaling is initiated through two different pathways, the MyD88-dependent pathway and the MyD88-independent pathway, which involves TIR-domain-containing adaptor-inducing interferon-β (TRIF) and the TRIF-related adaptor protein [39].

There have been numerous studies elucidating the role of TLR4 in NASH. Increased serum LPS levels are detected in patients with NASH and in animal models of NASH [49, 50], suggesting increased TLR4 activation. Furthermore, fructose, which is abundant in the Western diet, results in increased hepatic steatosis and inflammation potentially by causing increased intestinal bacterial overgrowth and intestinal permeability resulting in increased TLR4 signaling [51]. In MCD and HFD-induced steatohepatitis and fibrosis, TLR4 deficiency attenuates steatohepatitis [9, 52, 53]. In the MCD model, there is increased susceptibility to LPS stimulation, further implicating the TLR4 pathway in being involved in the pathogenesis of NASH [42]. MD2 knockouts also display a partial protection from MCD diet-induced steatohepatitis [52]. Both TLR4 and MD2 knockout mice display reduced NADPH oxidase activation and lipid peroxidation compared to WT mice, suggesting reduced free radical levels and improved lipid content. Furthermore, it has been suggested that TLR4 activation and sensitization of HSCs may be the link between inflammation and fibrosis in NAFLD and other forms of chronic liver disease, as HSCs are involved in the regulation of the extracellular matrix and tissue remodeling [54].

#### TLR9

TLR9 is expressed in intracellular vesicles and recognizes bacterial/viral DNA and CpG DNA. TLR9 ligands directly activate DCs, macrophages, and B cells, and they result in a strong T helper 1 (T_H_1) response. In murine NASH models, there are elevations in bacterial DNA in the plasma, which is a ligand for TLR9 [44]. The same group showed that TLR9 deficiency attenuates CDAA diet-induced steatohepatitis and fibrosis, suggesting a critical role of TLR9 in NASH [44].

### NOD-like receptors

The NLRs are a family of intracellular pattern-recognition receptors that are involved in the innate immune response to microbes by recognizing PAMPs (Fig. 2). NLRs also play an important role in recognizing host-derived signals, known as DAMPs. The NLR family is characterized by the presence of a central nucleotide-binding and oligomerization domain, flanked by C-terminal leucine-rich repeats and N-terminal caspase recruitment or pyrin domains. Further details of NLR complexes and their molecular structure are described elsewhere [55]. To date, four NLR prototypes have been identified, NLRP1 (NALP1), NLRP3 (NALP3, cryoporin), NLRC4 (IPAF), and AIM2 [56].

NLRs are part of intracellular multi-protein complexes known as inflammasomes that are assembled and activated following cellular infection or stress and result in inflammation. Inflammasome activation is thought to involve two steps, in which the first signal upregulates inflammasome expression and is generally from TLR activation, and the second step involves a ligand triggering inflammasome activation [56]. Inflammasome activation occurs in innate immune cells, including macrophages, neutrophils, and DCs, as well as non-immune cells, including hepatocytes, HSCs, endothelial cells, and myofibroblasts [57]. Furthermore, inflammasome activation results in the production of proinflammatory cytokines, including IL-1β and interleukin-18 (IL-18) [55]. The importance of the contribution of each cell type has not been entirely elucidated; however, one study showed that IL-1β or IL-1α depletion in hepatocytes, but not bone marrow cells, resulted in protection from diet-induced steatohepatitis [58].

IL-1β signaling plays an important role in NASH. IL-1R knockout mice showed attenuated liver steatosis, injury, and fibrosis following either a CDAA or HFD feeding [59]. In contrast, a different group found that both IL-1R knockout mice and WT mice treated with IL-1R antagonist (IL-1Ra) that received an MCD diet showed decreased liver steatosis but no improvement in inflammation or fibrosis (Csak et al., unpublished data). IL-1Ra-deficient mice showed severe hepatic steatosis and fibrosis on an atherogenic diet compared to WT mice fed the same diet [60]. Similarly to the IL-1R knockout mice, IL-1β knockout mice displayed attenuated hepatocellular damage, steatosis, and fibrosis following an atherogenic diet feeding [58]. The same group found that IL-1α knockout mice also displayed attenuated liver damage and fibrosis after an HFD feeding.

Thus far, mainly NALP3 has been found to have a potential role in the development of NASH. Further studies need to be completed with the other NLRs to determine whether they have a role in NASH.

#### NALP3

The NALP3 inflammasome is the most widely studied inflammasome and consists of the NLRP3 scaffold, the apoptosis-associated speck-like protein containing a caspase recruitment domain (ASC) adaptor, and the effector molecule, a proinflammatory protease, pro-caspase-1.

NALP3 is involved in antibacterial, viral, fungal, and parasitic immune responses [61]. Components of the NALP3 inflammasome and resultant caspase-1 activation are seen in the livers of mice fed both a HFD and a MCD diet [52, 62]. Increased expression of NALP3 and caspase-1 is also seen in the liver of humans with NASH [52], and the levels of expression in adipose tissue are directly correlated with the extent of type 2 diabetes mellitus in obese individuals [62]. Inflammasome upregulation has been found to occur in innate immune cells [56] as well as in primary hepatocytes of MCD diet-fed mice [52], suggesting that there are multiple cell types in the liver that undergo inflammasome activation and contribute to inflammation.

Mice lacking NALP3, ASC, or caspase-1 have been shown to display a decrease in weight gain and fat mass, and improved insulin resistance following a HFD feeding. However, there were differences among the three knockouts, with ASC knockout mice displaying decreased hepatic steatosis and liver triglyceride levels, whereas NALP3 and caspase-1 knockout mice did not show this change [63]. In contrast, a different group showed that ASC knockout mice are prone to steatosis, obesity, and increased glucose resistance [64]. This group suggested that the difference they find is due to alterations in the intestinal microbiome.

#### NLRP6 and NLRP12

NLRP6 and NLRP12 have mainly been studied in the context of colonic microbiota and inflammation [61]. NLRP6 knockout mice co-housed with WT mice display increased hepatic inflammation. On the other hand, NLRP12 mice co-housed with WT mice did not display any change in hepatic pathology. The group concludes that a transmissible colonic microbiota present in inflammasome-deficient mice results in NASH in the co-housed WT mice [64].

### RLRs and viral infections

Patients with NASH have been shown to have accelerated progression of liver disease and fibrosis and decreased efficacy of treatment with antiviral therapy in the setting of co-infection with hepatitis C virus (HCV) and human immunodeficiency virus [65, 66].

The mechanism behind the increased susceptibility to HCV infection in the setting of NASH has been attributed to a defect in the TLR3 signaling pathway. HCV is recognized in its dsRNA form by TLR3 and the RLRs, which include helicase receptors retinoic acid-inducible gene I (RIG-I) and melanoma differentiation-associated gene 5. RLRs are molecules that sense viral RNA molecules, resulting in the triggering of a danger signal. The adaptor mitochondrial antiviral signaling protein (MAVS) associates with the outer mitochondrial membrane and is crucial for inflammatory cytokine and interferon production after dsRNA binding to the helicases [67]. Whether endogenous RNA triggers RLR activation in NASH has yet to be studied.

Our group has shown that in the setting of MCD diet-induced steatohepatitis, mice challenged with the synthetic dsRNA, poly(I:C) show an inability to mount an efficient anti-viral response as demonstrated by impaired type I IFN production [16]. This deficiency was found to be secondary to dissociation of MAVS from the mitochondria to the cytosol and necrosis. Despite a decrease in inflammatory cytokine production, there was still an increase in liver damage in mice with MCD-diet-induced steatohepatitis.

### Microbiome

**Fig. 3:**
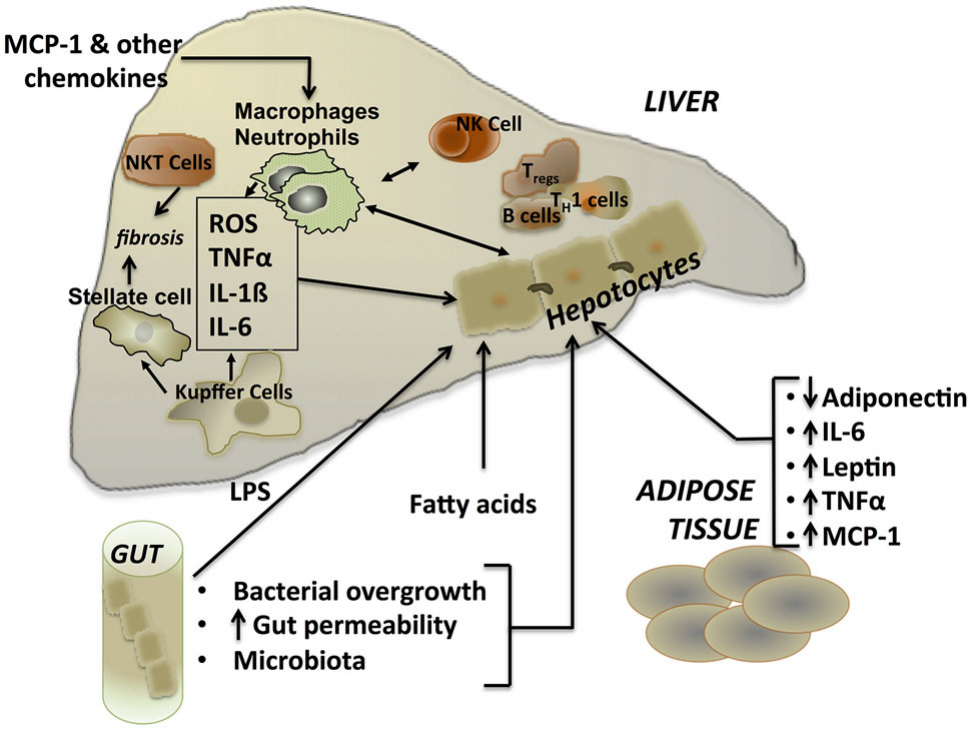
Cross-talk in NASH. The immune and inflammatory response in NASH involves an interaction among the liver, gut, and adipose tissue. Various adipokines, including adiponectin, interleukin-6 (IL-6), leptin, tumor necrosis factor α (TNFα), and monocyte chemoattractant protein-1 (MCP-1), are released by adipocytes and macrophages in adipose tissue and contribute to inflammation in the liver. Increased gut permeability, bacterial overgrowth, and changes in microbiota can result in the production of LPS, fatty acids, and other factors that contribute to liver inflammation and steatosis

Recently, the role of the intestinal microbiota and probiotics in liver diseases has been a popular area [64, 68]. It is well known that patients with NAFLD have increased intestinal permeability and a higher incidence of bacterial overgrowth in the small intestine compared to patients with normal livers [49]. The translocation of bacterial components promotes hepatic inflammation through TLR signaling and KC activation [9] (Fig. 3). An increase in TLR agonists in the intestine has been shown to drive the progression of NASH in WT mice [64].

In mice lacking various inflammasome components, a significant expansion of the intestinal bacteria Porphyromonadacae was detected following a HFD and MCD diet feeding. These bacteria have been implicated in the development and progression of metabolic syndrome in both mice and humans. Treatment with antibiotics abolished the bacteria [64, 69]. Rats fed a HFD and treated with probiotics displayed a change in intestinal microflora and a reduced level of hepatic cholesterol and triglycerides [70]. Treatment with the probiotic VSL no. 3 in mice fed an MCD diet resulted in attenuation of fibrosis without protecting from inflammation or steatosis [71].

## Danger-associated molecular patterns

DAMPs are endogenous molecules released from damaged cells that trigger sterile inflammation [72]. Sterile inflammation is a response that occurs in all tissues in response to injury and cellular damage. In NASH there is a chronic state of sterile inflammation resulting from the presence of DAMPs. Similarly to PAMPS, DAMPs activate both the innate and adaptive branches of the immune response (Fig. 1). A wide variety of DAMPs have thus far been identified in many different diseases and are reviewed elsewhere [73]. DAMPs activate PRRs, of which the best characterized are TLRs (Fig. 2). In NASH, it has been a challenge to show an increase in the levels of serum DAMPs, and further research is needed to elucidate whether DAMPs that play a role in other diseases also contribute to the pathogenesis of NASH [73]. Some of the DAMPs that have been identified, such as high-mobility group-1 (HMGB1) and FFAs, signal through several TLRs and, in the case of FFAs, a NLR.

### Toll-like receptors (TLRs)

#### TLR2

It has not yet been determined whether there are DAMPs that directly activate the TLR2 ligand in NASH. Stimulation of TLR2 in KCs with a TLR2 ligand in the presence of the FFA palmitic acid (PA) results in inflammasome activation and increased IL-1β production, suggesting a cross-talk between PAMPs and DAMPs [44]. It has been hypothesized that HMGB1 may also activate TLR2 in addition to TLR4. As discussed earlier, there are opposing phenotypes published by different groups in the setting of TLR2 knockout mice in a diet-induced steatohepatitis model, suggesting that differences in intestinal microflora could be responsible for TLR2 ligand production [44].

#### TLR4

A variety of DAMPs have been described that activate TLR4, including HMGB1, hyaluronic acid, heat shock proteins, defensins, and fibrinogen [41]. The role, if any, of most of these DAMPs has not yet been conclusively determined in NASH.

HMGB1 is a highly conserved nuclear protein that facilitates regulatory protein binding to DNA [74]. It is constitutively expressed by most cells and is released upon cell injury or death. Yanai et al. [75] describe HMGB proteins as acting as universal intracellular sensors of cytosolic nucleic acids. For example, HMGB1 is released from necrotic cells leading to KC and monocyte stimulation by acting as an endogenous TLR4 ligand either alone or with other molecules, such as endotoxin or ssDNA [76]. Furthermore, increased hepatocyte expression and release of HMGB1 triggers TLR4 and MyD88 signaling and inflammation in response to FFA infusion or HFD [53]. The interaction between HMGB1 and FFAs in TLR4 signaling in NASH is yet to be studied in depth. It has not yet been determined whether HMGB1 directly binds to TLR4 or forms complexes with other molecules.

FFAs have also been described to contribute to the pathogenesis of NASH [1, 77]. FFA levels have been reported to be elevated in mice with HFD-induced, MCD diet-induced, or leptin-deficiency diet-induced steatohepatitis [78–80]. Saturated FFAs result in the activation of the TLR4 pathway in adipocytes and macrophages resulting in inflammation through an unclear mechanism [73]. However, it does not appear that FFAs are ligands for TLR4 in either KCs or HSCs [44, 81].

#### TLR5

Although not expressed in mammalian livers, TLR5 knockout mice have altered gut flora. These mice develop obesity, steatosis, and insulin resistance secondary to hyperphagia. Transmission of the gut flora from these mice to WT mice resulted in transmission of the disease phenotype [82].

#### TLR9

Bacterial DNA is often detected in the blood of both rodents and humans with chronic liver diseases [83, 84]. DNA from dying hepatocytes in NASH can act as an endogenous ligand for TLR9 [85]. The DNA from apoptotic cells is modified by caspase-activated DNAse-mediated cleavage and methylation and oxidative damage, resulting in the ability to activate TLR9 [85]. The combination of both exogenous and endogenous bacterial DNA could potentially lead to an exponential increase in TLR9 activation in NASH. Mitochondrial dysfunction has also been implicated in the pathogenesis of NASH, resulting in increased oxidative stress, cytokine production, and cell death [86]. Mitochondrial DNA, which resembles bacterial DNA in having non-methylated CpG motifs, released by injured cells results in the activation of neutrophils through TLR9 signaling. Furthermore, HMGB1 has also been described to interact with TLR9, likely involved in the signal response to CpG DNA [75], but its role in NASH is not yet clear.

### NOD-like receptors (NLRs)

#### NALP3

The NALP3 inflammasome can be activated by a variety of host-derived molecules. These activators include extracellular ATP, hyaluronic acid, amyloid-β, and uric acid crystals [55].

Both experimental and clinical studies have shown a link between IL-1β and IL-18, which are produced by inflammasome activation, to the development of metabolic syndrome [87, 88]. The underlying mechanism has not yet been elucidated.

Obesity has been shown to result in adipocyte hypertrophy and preferential M1 macrophage infiltration [89]. The NALP3 inflammasome is activated in these macrophages by the saturated fatty acids PA and lipotoxic ceramics [62, 90]. Hepatocytes treated with PA displayed upregulation of the NALP3 inflammasome and caspase-1 activation. Hepatocytes stimulated with PA have been shown to activate liver mononuclear cells, suggesting that hepatocytes can transfer inflammasome activation to surrounding immune cells [52]. Furthermore, pre-treatment of hepatocytes with PA resulted in sensitization to stimulation with LPS and a significantly higher level of caspase-1 than treatment with either PA or LPS alone [52]. In addition to leading to inflammation, fatty acids also induce apoptosis and the subsequent release of DAMPs. The release of mitochondrial DNA into the cytosol in response to LPS and ATP stimulation is dependent on NALP3 and is necessary to achieve robust caspase-1 activation and IL-1β production [91]. Mitochondrial DNA appears to also act by directly binding to and activating NALP3 [92] and likely contributes to the pathogenesis of NASH.

## Role of adipose tissue

Adipose tissue is a highly active tissue that has both endocrine and immune functions, and has been found to have a role in mediating disease. In obesity, there is dysfunctional adipose tissue resulting from hypertrophic adipocytes and macrophage infiltration into adipose tissue [93]. The liver is an important target for adipose tissue, and its dysregulation in obesity can contribute to steatohepatitis (Fig. 3). The increase in FFAs seen in obesity results in TLR4 activation in adipocytes and macrophages, resulting in inflammation. Furthermore, there is an increase in circulating FFAs secondary to an increase in adipose FFAs, resulting in an increase in portal FFAs, thus contributing to NAFLD and eventually NASH [94]. Activation of adipose tissue macrophages also results in ectopic fat deposition in the liver.

Hypertrophic adipocytes behave similarly to macrophages and produce adipokines similarly to foam cell fat-loaded macrophages in arterial plaques [6]. The production of insulin-sensitizing adipokines is reduced, such as adiponectin, resulting in decreased insulin sensitivity. Overexpression of adiponectin in genetically obese leptin-deficient ob/ob mice results in a reversal of the diabetic phenotype and reduced liver fat content, despite retaining the obese phenotype, suggesting that adiponectin plays a critical role in preventing both local and systemic inflammation [95].

Other adipokines that have a proinflammatory role are the cytokines interleukin-6 (IL-6) and TNFα as well as the chemokine monocyte chemoattractant protein-1 (MCP-1). IL-6 levels are elevated in the serum and adipocytes of obese patients, and the level of expression is significantly higher in adipose tissue than in liver, suggesting that adipose tissue is the main source of IL-6 in NASH [96, 97]. Furthermore, TNFα expression is profoundly increased in adipose tissue of obese patients [96]. Adipose tissue-derived IL-6 and TNFα likely target the liver, as exposure to both of these cytokines has an effect on hepatic insulin resistance [98]. IL-6-deficient mice fed a MCD diet or HFD develop worsening inflammation and liver damage [99, 100]. However, blockade of IL-6 signaling by a neutralizing antibody against the IL-6 receptor resulted in enhanced liver steatosis and improved liver injury in MCD-fed mice [101]. The balance in the levels of IL-6 appears to play an important role in the development of liver injury.

## Conclusions

NAFLD is a disease that encompasses a wide range of pathology, from steatosis to steatosis with inflammation to fibrosis; it is commonly seen in the presence of the metabolic syndrome. It is clear that there are complex immune and inflammatory pathways that result in the progression of NASH, involving signaling in various cell types that are stimulated by PAMPs and DAMPs as well as interaction between different cell types and tissues. While there has been a significant amount of research examining the immune and inflammatory pathways in NAFLD, many questions remain. The reason why certain people develop steatohepatitis and progress to cirrhosis while others only develop steatosis has not yet been determined. There are likely undiscovered PAMPs and DAMPs that contribute to NAFLD, as well as unknown interactions between various PAMPs and DAMPs. Furthermore, understanding cell specificity in both NLR and TLR activation in NASH is an area that deserves further investigation and could be important as a target for future therapeutics. In addition, more studies looking at the interactions of the components of the adaptive immune system are warranted.

Despite the significant advancement in knowledge about the development of NAFLD, a dearth of effective treatments remains, aside from weight loss and treating associated conditions, such as hyperlipidemia and diabetes. Continuing to elucidate the mechanisms and pathogenesis behind the progression of NAFLD is critical to being able to design therapeutic interventions that can directly target the liver.
